# Development of a novel immunocompetent murine tumor model for urothelial carcinoma using in vivo electroporation

**DOI:** 10.1038/s41598-024-77178-z

**Published:** 2024-10-27

**Authors:** Stefan Soleder, Nicolas Gengenbacher, Carolin Mogler, Markus Eckstein, Anja Runge, Maximilian C. Kriegmair, Hellmut G. Augustin

**Affiliations:** 1 Division of Vascular Oncology and Metastasis Research, German Cancer Research Center Heidelberg (DKFZ), Heidelberg, Germany; 2 European Center for Angioscience (ECAS), Medical Faculty Mannheim, Heidelberg University, Ludolph-Krehl-Str. 13-17, 68167 Mannheim, Germany; 3 Department of Pathology, Technical University of Munich, Munich, Germany; 4 Institute of Pathology, University Hospital Erlangen, Friedrich-Alexander-Universität Erlangen- Nürnberg, Erlangen, Germany; 5 Department of Urology, University Hospital Mannheim, Mannheim, Germany

**Keywords:** Bladder cancer, Cancer models, Cancer models

## Abstract

A lack of advanced preclinical mouse tumor models impedes the progress in urothelial carcinoma research. We present here a novel fast, robust, reliable, and highly reproducible model for the genetic induction of bladder cancer in immunocompetent mice. Different sets of oncogenic transposons (*Cmyc*, *Kras*) and Cre drivers were transfected into the murine bladder wall of two different genetic backgrounds (*Trp53*^*fl/fl*^ and *Braf*^*V600E*^, *Pten*^*fl/fl*^, *Ctnnb1*^exon3-fl/fl^). Transfection was carried out using in vivo electroporation of the bladder after surgical exploration and transmural or transurethral intravesical plasmid injection. Up to 100% of animals developed urothelial carcinomas of the bladder. Time to tumor onset ranged from 16 to 97 days with a median of approximately 23 days in the fastest groups. Histological examination identified orthotopic urothelial carcinomas in most cases, in some experimental groups up to 100%. The resulting tumors were highly invasive and often metastatic. Metastases were found in up to 100% of tumor bearing mice per group. Taken together, this study establishes the proof-of-principle that in vivo electroporation can be versatilely employed as a reliable, fast, and robust method for the highly reproducible induction of urothelial carcinomas in the murine bladder wall. This novel murine tumor model could pave the way towards more easily modelling subtype specific urothelial carcinomas in mice.

**Supplementary Information:**

The online version contains supplementary material available at 10.1038/s41598-024-77178-z.

## Introduction

Urinary bladder cancer (UBC) is the 10th most common cancer entity worldwide, with 7.4 cases and a mortality rate of 2.7 per 100,000 people in 2020^1,2^. Muscle invasive bladder cancer (MIBC) accounts for approximately 25% of cases and has a strong tendency for metastasis and an overall poor prognosis^3^. Therapeutic advances for metastasized bladder cancer have long failed to materialize. Only recently have immunotherapy and targeted agents been approved for treating metastasized UBC^4,5^. Current challenges for clinical, preclinical and translational research are identification of methods to better stratify patients to a suitable treatment as well as identifying ideal sequences or combinations of therapies^6,7^.

The historically slow rate of progress in UBC therapy may at least in part be attributed to the lack of well-established mouse models for urothelial carcinoma^8,9^. The absence of genetically engineered models (GEMs) is especially striking in the case of bladder cancer, as GEMs have been crucial in advancing research in other tumor entities^8–11^. Previous mouse model studies most commonly employed the carcinogen BBN to induce tumors in the bladder wall. BBN is representative of smoking as the primary pathogen causing bladder cancer but has overall slow tumor growth typically taking 3–5 months^12–16^.

In vivo electroporation for gene transfer has been shown to be a viable method in many organs^17–23^, including the murine bladder^24^. The aim of this study was therefore to develop a new genetically engineered tumor model for UBC in immunocompetent mice. We used the available biomolecular tools of Cre recombinase and Sleeping Beauty transposase in conjunction with in vivo electroporation to achieve specific genetic alterations locally in the bladder mucosa in order to generate orthotopic UBC with a distinct genetic starting point. We describe here as proof-of-principle a novel method for inducing different types of UBC in mice and report first results generated using this method.

## Results

### Reliable and fast induction of orthotopic urothelial cell carcinoma

**Fig. 1:**
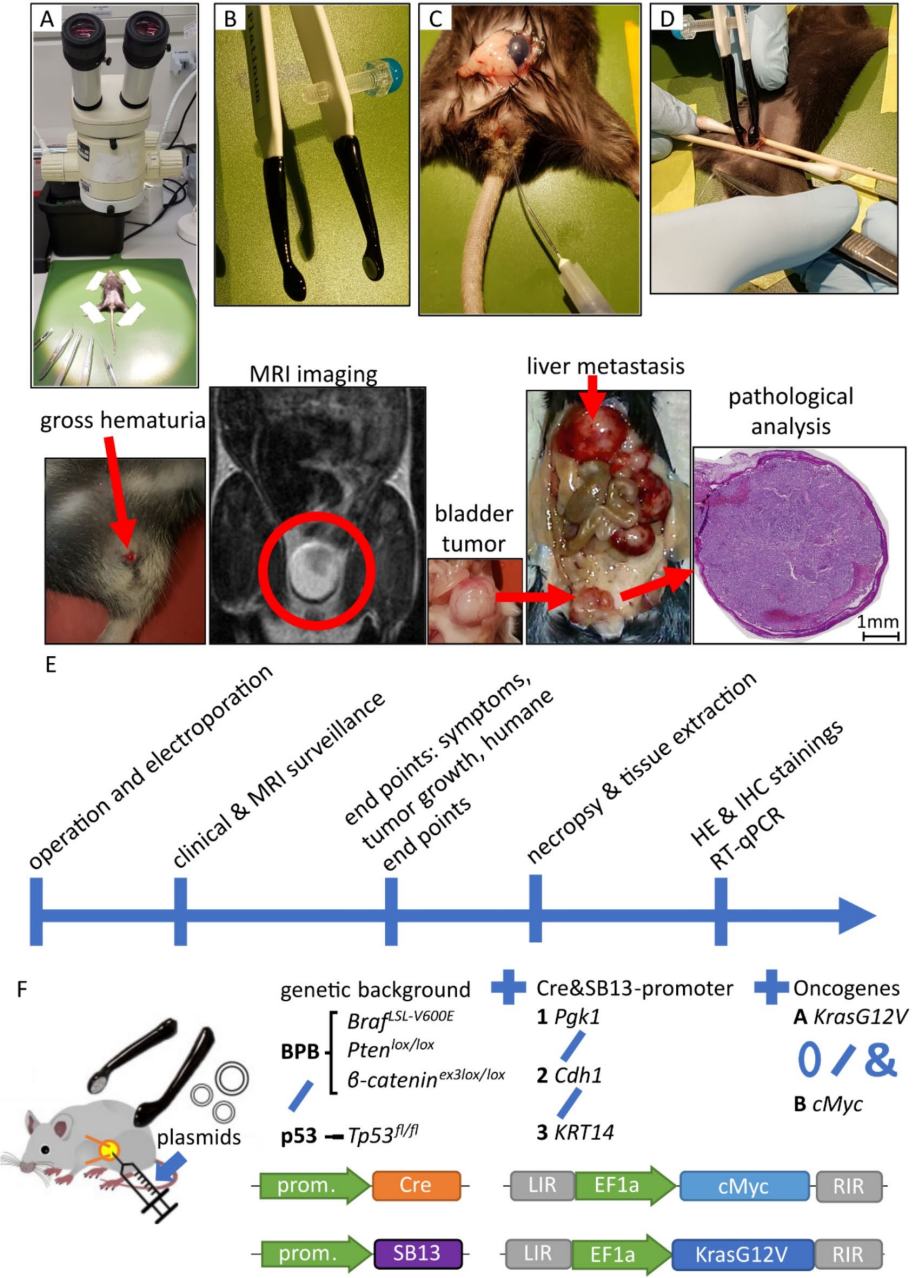
Key features of operation procedure and experimental protocol. (**A**) Setup for surgical procedure; (**B**) platinum coated electrode tweezers for applying electrical current; (**C**) transurethral injection using tubing mounted onto injection needle inserted through urethra, shown with blue dye; (**D**) stabilization of bladder, closure of urethral outlet with tweezers and application of current; (**E**) process steps of experimental protocol with exemplary images; (**F**) schematic of genetic backgrounds, promoters and plasmids used: 0 denoting use of neither, / denoting choice of one and & concurrent application of options.

A first cohort of 8 experimental groups was set up using the approach outlined in Fig. 1 to assess the viability of the experimental approach. The 8 groups shown resulted from using all possible combinations of the two backgrounds (*Trp53*^*fl/fl*^ abbr. p53 and *Braf*^*V600E*^, *Pten*^*fl/fl*^, *Ctnnb1*^exon3-fl/fl^ abbr. BPB) with *Kras*,* Cmyc* simultaneously, alone or no oncogene, with Cre and SB13 always under a *Pgk1*-promoter. Employing this strategy, we successfully generated tumors in 7/8 groups. Mice developed tumors in 62,5% (BPB + Cre only) to 100% (p53 + *Kras*, BPB *Cmyc*, BPB *Kras*, and in p53 *Kras + Cmyc* all remaining mice after euthanization of one mouse postoperatively) of cases. Histological analysis confirmed that the generated tumors were in most cases urothelial cell carcinomas, ranging from 40% (p53 + *Cmyc)* up to 100% of experimental animals (p53 + *Kras + Cmyc*) and of those tumors shown to be urothelial cell carcinomas, we detected the primary tumor in the bladder in almost all cases (Supp. Table 1). Median time to tumor take varied between groups from 29 days (p53 + *Kras + Cmyc*) to 83 days (p53 + *Cmyc*). The p53 group receiving only Cre showed no tumor growth at the predefined maximum observation period of 250 days after electroporation as expected. Tumor penetration in total was 74,4%. Of all mice, 55,3% developed orthotopic tumors, whereas ectopic tumors were found in 19,1% of mice, with no evidence of bladder involvement.

Tumor behavior strongly resembled invasive urothelial cell carcinomas. Gross hematuria as important clinical symptom in humans was observed in 9,7% of tumor-bearing mice in the first cohort. Metastasis occurred in up to 100% of mice developing an orthotopic urothelial carcinoma (Supp. Table 1). The overall most successful group was the p53 Cre/SB13 + *Kras + Cmyc* group, as it showed 100% orthotopic urothelial carcinomas with the fastest time to tumor take with a median of 29 days after electroporation. The combination of both oncogenes was, thus, used in all subsequent experiments.

Surgery-associated morbidity and mortality was low. In both cohorts, a total of 3 mice out of 139 had to be euthanized due to bad clinical recovery after surgery. The surgical approach outlined above was, thus, safe and technically simple to carry out. The gene expression levels of the targeted oncogenes and tumor-suppressor genes were as expected in the p53 group with higher expression of *Kras* and *Cmyc* and deletion of *Trp53* (Supp. Figure 3). In the BPB group, *Kras* showed normal expression levels in most tumors, despite the same transfection solution, whereas *Cmyc* was overexpressed. *Pten* was deleted as desired, the genetic alteration of Ctnnb1 (exon3 deletion) did not lead to changes in expression levels (Supp. Figure 3).

**Fig. 2:**
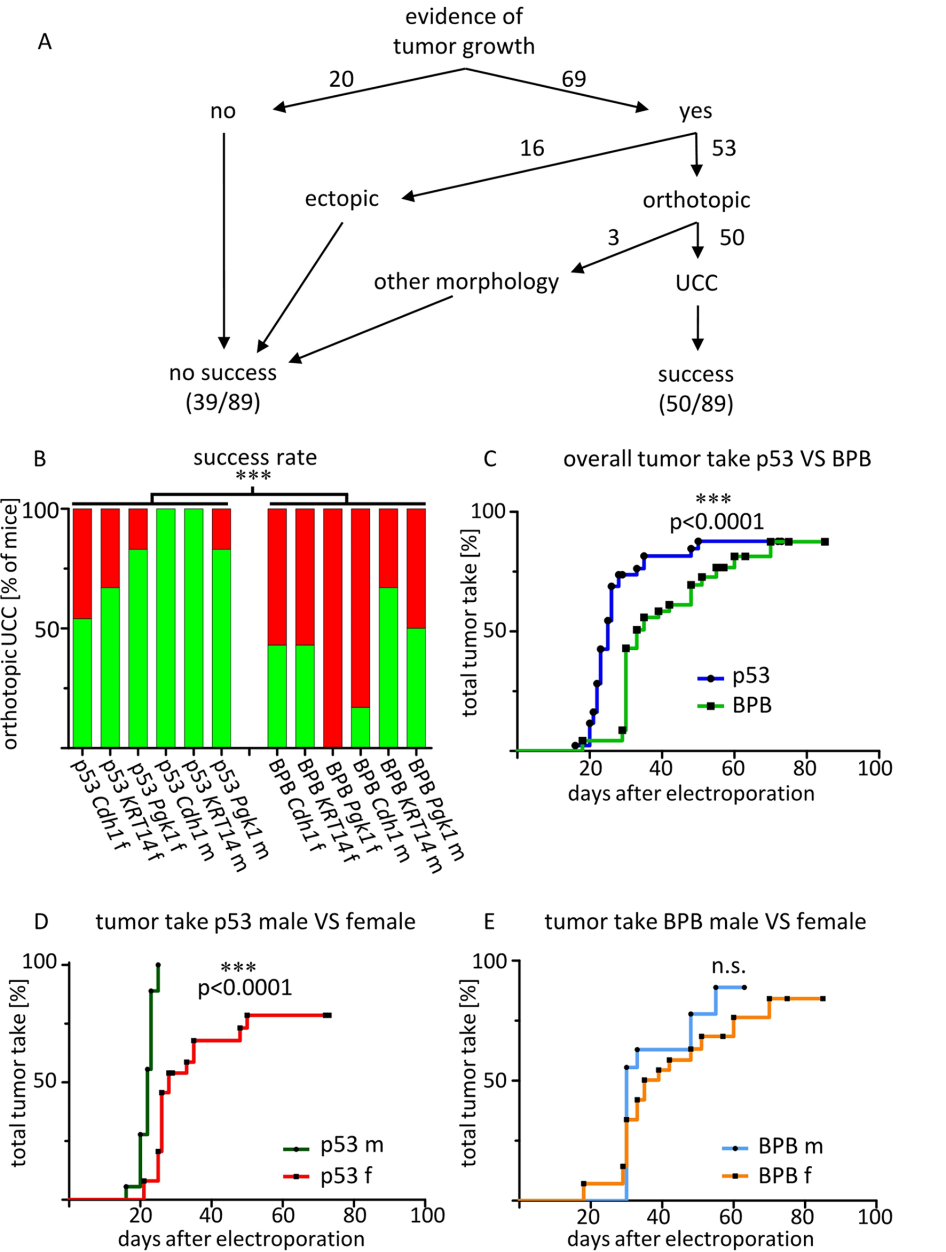
Rapid and robust tumor take of orthotopic urothelial cell carcinoma across groups with differences between transgenic backgrounds and sexes. An overall success rate of the second cohort for growth of any urothelial carcinoma variant found orthotopically in the bladder (“success”, green) versus all other outcomes (red), based on pathology consensus (f = female, m = male). Statistically significant difference between *Trp53*^*fl/fl*^ (abbr. p53) and *Braf*^*LSL−*^*V600E*,* Pten*^*lox/lox*^, *Ctnnb1*^*ex3lox/lox*^ (abbr. BPB) genetic background, Fisher‘s exact test *p* = 0.0002. From left to right *n* = 13, 6, 6, 6, 6, 6 (for p53), 14, 7, 7, 6, 6, 6 (for BPB); B overall tumor take rate curves, i.e. time to first tumor detection and euthanasia with proven tumor growth at necropsy or histology, for p53 VS BPB genetic background in the second cohort, log-rank test *p* < 0.0001, *n* = 43 for p53, *n* = 46 for BPB; C tumor take comparison between sexes for p53 genetic background, second cohort, *n* = 18 for male, *n* = 25 for female; D tumor take comparison between sexes for *Braf*^*LSL−V600E*^, *Pten*^*lox/lox*^, *Ctnnb1*^*ex3lox/lox*^ genetic background, second cohort, *n* = 18 for male, *n* = 28 for female.

Based on the findings of the first cohort, the main experiment was carried out in a second cohort using both oncogenes, i.e. *Kras + Cmyc* in all groups. To increase specificity for the urothelium and avoid primary peritoneal carcinoma or muscle wall sarcomas upon plasmid leakage, epithelium-specific promoters were generated and used (*KRT14-Cre + SB13*,* Cdh1-Cre + SB13)*, resulting in 6 genetic groups for 2 genders (Fig. 1, Supp. Table 1). All groups developed tumors. Generation of orthotopic urothelial carcinomas (termed ‘success’) was achieved in up to 100% of mice (p53 *KRT14-*Cre male and p53 *Cdh1-* Cre male groups) (Fig. 2A). The average success rate across all groups was 56,2%. Overall tumor take was 77,6% of mice, 59,6% orthotopically and 18,0% ectopically. The p53 groups developed tumors significantly faster (25 days vs. 33 days, *p* = 0.0001) and more reliably (HR 2.9, 95% CI 1.7-5.0; *p* = 0.0002) than the BPB groups (Fig. 2A-C), with the fastest median times to tumor take occurring in the p53 male *Pgk1*-Cre/SB13 + *Kras + Cmyc* group (20 days), p53 male *Cdh1*-Cre/SB13 + *Kras + Cmyc* group (22,5 days) and p53 male *KRT14*-Cre/SB13 + *Kras + Cmyc* group (23 days) (Supp. Table 1). Hematuria was found in 11,2% of tumor-bearing mice, urinary retention caused by obstructive tumor growth developed in 17,4% of mice. A significant difference in tumor take was observed between sexes in the p53 background with males / transmural injection reaching ‘success’ more often than in females / catheterization (Fishers’ exact test p = 0.0284), but not so in the BPB background. Male mice in the p53 background also developed tumors significantly faster than females. The time to first tumor detection in MRI or clinically with subsequent proof at necropsy or histology was 22 days vs. 28 days (p < 0.0001) respectively (Fig. 2D). This was not the case in the BPB background (Fig. 2E).

**Fig. 3:**
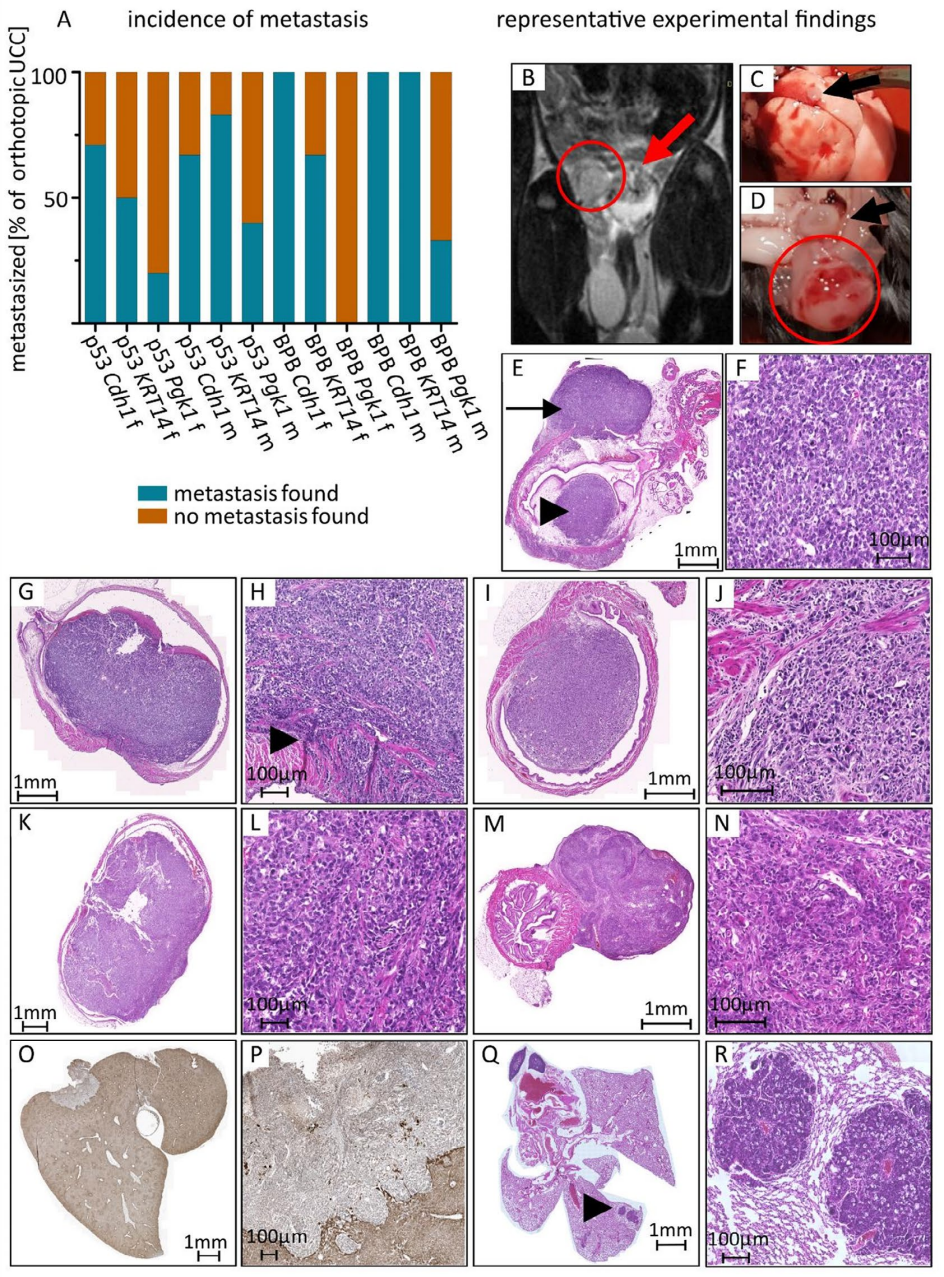
Macroscopic and microscopic analysis show highly invasive and metastatic tumor behavior. A proportion of metastasized tumors in relation to all orthotopic UCC, from left to right, number of orthotopic UCC *n* = 7, 4, 5, 6, 6, 5 (for p53), 6, 3, 0, 1, 4, 3 (for BPB); B MRI scan showing intraluminal tumor growth into the bladder wall (arrow) and peritoneal metastasis (circle); C macroscopic image of lung metastasis (arrow) at necropsy; D macroscopic image of peritoneal metastasis (circle) at necropsy, bladder marked with arrow; E, F HE stained microscopic image of invasive tumor growth into submucosa reaching muscular bladder wall (arrowhead) and local peritoneal metastasis on the outside bladder wall (arrow), histologically conventional type of UCC with intraluminal and extraluminal growth; G,H HE staining of intraluminal tumor invading the muscular bladder wall (arrowhead), conventional carcinoma; I,J HE staining of muscle invasive tumor with sarcomatoid and conventional parts; K,L HE staining of muscle invasive sarcomatoid urothelial carcinoma; M,N HE staining of tumour with squamous features; O,P Pan-Cytokeratin staining of liver metastasis; Q, R HE staining of lung metastases.

**Fig. 4:**
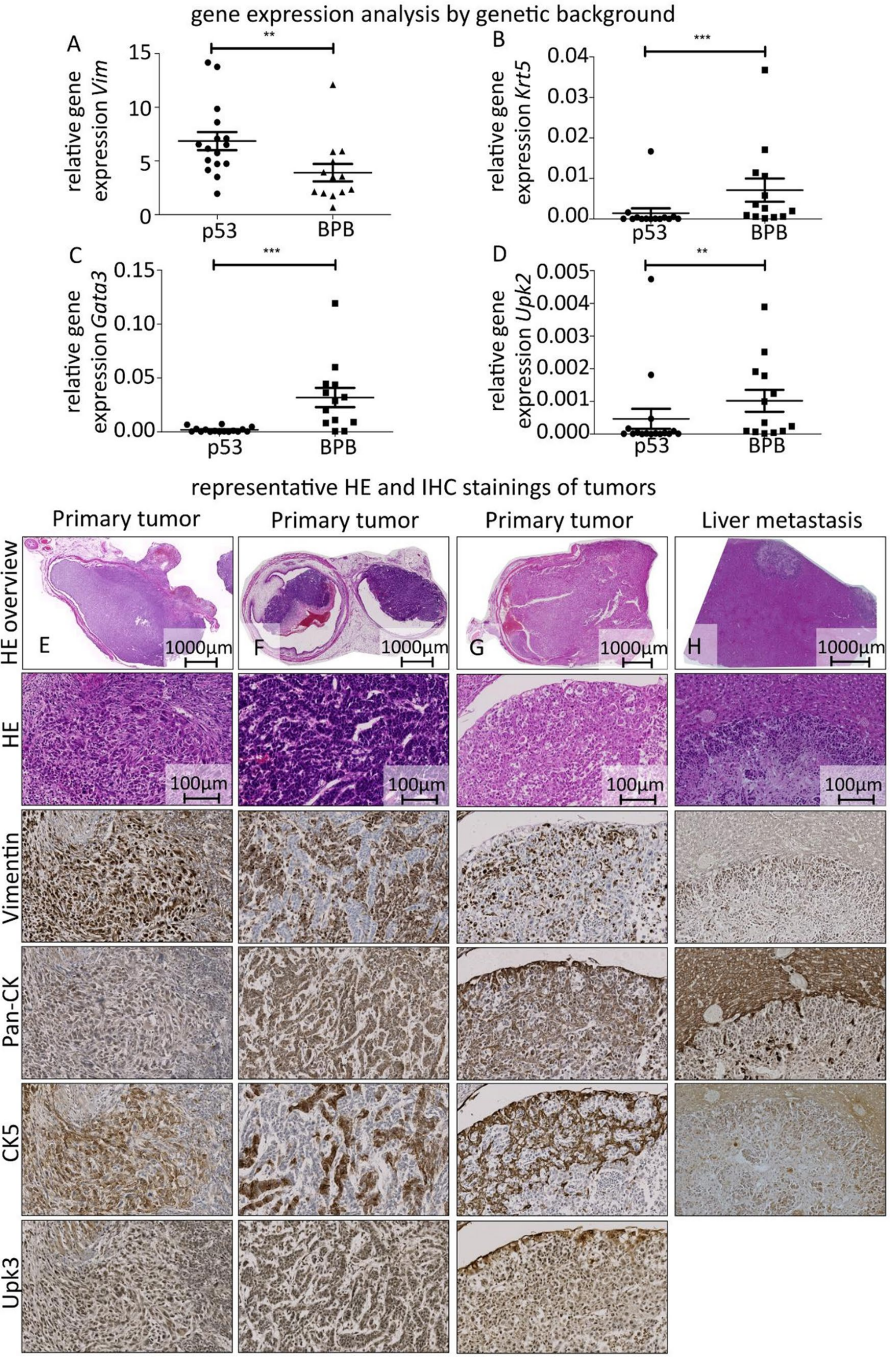
Histopathological and gene expression analysis confirms urothelial cell carcinoma biology and shows differences between genetic backgrounds. A-D Comparison of relative gene expression of between genetic backgrounds (*Trp53*^*fl/fl*^ abbr. p53, *n* = 16 samples, and *Braf*^*LSL−V600E*^, *Pten*^*lox/lox*^, *Ctnnb1*^*ex3lox/lox*^ abbr. BPB, *n* = 13 samples) for different target genes in the second experimental cohort, bars indicating mean ± SEM: C *Vim*, statistically very significant difference in gene expression, Mann-Whitney test: *p* = 0.0080; D *Krt5*: *p* = 0.0006; E *Gata3*: *p* = 0.0001; F *Upk2*: *p* = 0.0078; E-H representative image panels, 3 different tumors and liver metastasis in respective stainings showing the same tumor area; E mixed sarcomatoid and conventional carcinoma with muscle invasion; F muscle invasive conventional urothelial carcinoma; G muscle invasive tumor showing different morphological forms; H liver with multiple metastases.

Muscle-invasive growth was present in most tumors (Fig. 3E-N, Supp. Table 1). Tumor size was frequently larger than the bladder of the mouse, making tumor staging difficult. The numbers given for muscle invasiveness represent tumors where muscle invasive growth was seen histologically (Supp. Table 1). Tissue sections frequently missed the exact point of muscle invasion due to organ size and resection or tissue section margins. The percentage in brackets is the total fraction of orthotopic UCC tumors showing histologic evidence of growth beyond the muscularis, where the exact point of entry into the muscularis was not found. Metastasis was frequent across groups (Fig. 3A, Supp. Table 1) and observed in the peritoneum, the lungs and the liver (Figs. 1E, 3B-R and 4H). All metastases detected were found at the same time as primary tumor detection, as mice were euthanized upon first tumor detection.

In conclusion, tumor growth was reliable, fast and resulting tumors were invasive and metastasized early. The p53 groups developed tumors most frequently, particularly in male mice. Using two oncogenes and the epithelium-specific promoters *KRT14* and *Cdh1* resulted in the most frequent growth of orthotopic urothelial carcinoma of all experimental groups.

### Tumor analysis validates urothelial tumor origin and reveals biological differences between genetic backgrounds

To characterize the resulting tumor biology and confirm the urothelial origin, histopathological analyses including IHC stainings and gene expression analyses were performed on all tumors. Gene expression patterns were consistent with urothelial cell carcinomas in humans. Immunohistochemistry revealed partially preserved cytokeratins, especially cytokeratin 5, with carcinoma cells being vimentin negative and tumor stroma vimentin positive (Fig. 4E-G). Histopathological analysis showed ‘conventional UCC’, ‘conventional UCC with sarcomatoid parts’ and ‘sarcomatoid UCC’ most commonly, but urothelial carcinomas with squamous features were also observed (Supp. Figure 2). Tumors of the ‘conventional UCC’ type frequently had preserved uroplakin 3 expression. Pathological analysis regarding morphology and diagnosis was concordant in 96,6% (86/89) of cases. Most tumors with no evidence of orthotopic growth in the bladder, i.e., where tumor was only found ectopically (e.g., peritoneal tumor) were also identified as urothelial carcinomas (Supp. Figure 2). In the first and second round respectively, 9 out of 9 and 13 out of 16 ectopic tumors were identified as urothelial carcinomas.

Gene expression analyses revealed a significant difference in gene expression for vimentin, keratin 5, *Gata3*, and uroplakin 2 between the genetic backgrounds (Fig. 4A-D). The p53 background mice expressed significantly higher levels of vimentin and significantly lower levels of keratin 5, *Gata3* and uroplakin 2 than the BPB background, indicating further dedifferentiation and remaining consistent with the faster tumor development in this background.

In conclusion, the resulting tumors in both groups were highly reminiscent of a basal-like urothelial bladder cancer. The BPB groups particularly resembled the human basal-squamous subtype^3,25–30^ with a strong mesenchymal component, showing high expression of keratin 5 and lacking *Gata3* expression while also being high in vimentin. The p53 groups showed some features of a neuroendocrine-like subtype: worse prognosis, *Trp53* knockout as defining mutation, further dedifferentiation, lower in *Krt5*,* Upk3* and *Gata3*, higher in *Vim*^25^. However, the data were not sufficient to clearly assign the tumors to subtypes. The results are in line with the expected tumor phenotype both in cases of *Trp53* mutations^31–33^ such as the one induced in the *Trp53*^*fl/*fl.^ background and in the case of our triple-transgenic model with several targets in the RTK/RAS/PI(3)K pathway^31^.

## Discussion

Versatile genetically engineered mouse models for bladder cancer to progress cancer research have been and still are sorely lacking^8–10^. A molecular classification for different subtypes of bladder cancer has recently been proposed^25^ and new models mimicking these are arising, albeit with some difficulty and limitations^34^. In vivo electroporation has recently been shown to be a viable approach of delivering plasmids into the bladder mucosa^24^.

We present here the first successful generation of orthotopic urothelial bladder carcinomas by genetic modification using in vivo electroporation. The experiments resulted in the rapid development of 2 biologically distinguishable urothelial carcinoma phenotypes depending on the genetic alterations introduced, both showing muscle invasion, local and distant metastasis as well as clinical features of urothelial carcinomas such as hematuria and urinary stasis. This method is a viable, robust and efficient way of generating urothelial carcinomas in mice.

The resulting tumors showed significant differences in their tumor biology and behavior, especially between the *Trp53*^*fl/fl*^ and the *Braf*^*LSL-V600E*^, *Pten*^*lox/lox*^, *Ctnnb1*^*ex3lox/lox*^ backgrounds. Tumors with a loss of *Trp53* generally show a more basal-like biology^31,35,36^. Consistent with this, the experimental groups using the *Trp53*^*fl/fl*^ background showed faster and more reliable tumor growth with a higher proportion of urothelial cell carcinomas and possibly similarities to the neuroendocrine-like subtype. The *Braf*^*LSL-V600E*^, *Pten*^*lox/lox*^, *Ctnnb1*^*ex3lox/lox*^ groups showed more markers of the newly described basal-squamous subtype. For generating basal-like UBC, we, thus, favored the combination of a *Trp53* knockout with the plasmid combination of added *Kras* and *Cmyc* and using an epithelium-specific promoter like *KRT14*. We favored this combination due to the faster tumor take and the higher rate of urothelial cell carcinomas. Gene expression analyses performed were not as in-depth as current RNAseq characterizations and we can therefore not definitely pinpoint tumor biology to one of the newly defined specific urothelial carcinoma subtypes. The obtained data would, however, argue towards a more basal-like biology in both groups. The lack of tumor take in the experimental group with a *Trp53*^fl/fl^ background receiving only Cre was also consistent with what has been described for a sole *Trp53* knockout in the literature^36^. Further modifications of the resulting tumors can be achieved by employing yet other plasmid combinations. For example, in vivo electroporation using more than four plasmids simultaneously achieving efficient transfection has successfully been done in other organs^19,37^. The surgical procedure is safe and easy to learn and carry out. The genetic alterations we aimed to introduce proved to be present in the p53 group tumors. In the BPB group tumors, expression levels of *Kras* remained mostly normal. The *Ras/Raf* pathways were altered in several ways in the BPB background mice, which might explain the different expression levels in the p53 groups and the BPB groups. We could not prove expression of altered proteins for *Ctnnb1* and *Braf*, due to difficulties identifying suitable antibodies. *Pten* deletion and *Cmyc* overexpression were successful. These differences may explain some of the biological differences. While we found some ectopic tumor growth without evidence of a primary lesion in the bladder, we attribute this to early metastasis of the tumor, as most ectopic tumors still were urothelial carcinomas (Supp. Figure 2).

Taken together, the new model is faster than existing urothelial carcinoma mouse models described in the literature, highly versatile and easily modifiable. It is (i) an orthotopic genetically modified model of bladder cancer, (ii) can be carried out in immunocompetent mice, (iii) eliminates the need for breeding to achieve a certain set of tumorigenic mutations while (iv) still having a defined genetic starting point, (v) overcomes limitations regarding urothelium-specific or time-specific genetic alterations, (vi) mimics clinical symptoms seen in humans and (vii) can be used safely with minimal surgery-related morbidity of experimental animals. These advantages over previously described mouse models for UBC make it an ideal platform for subtype-specific tumor models in the bladder, facilitating advances in urothelial carcinoma biology research and development of targeted therapeutics for different bladder cancer subtypes.

### Methods

Figure 1 provides an overview of the experimental setup. Supp. Table 1 shows an overview of all experimental groups and main results. Experiments were only counted as successful when tumors were concordantly identified as urothelial carcinoma inside the bladder independently by two pathologists (C.M., M. E.).

### Mouse lines and animal studies

Animal experimentation protocols were designed with regards to the 3Rs of animal studies (replace, reduce, refine). All experiments were carried out in adherence to the guidelines of the DKFZ institutional and governmental Animal Care and Use Committees and reviewed and approved by the Regierungspräsidium Karlsruhe (permit numbers G126/18 and extension/alteration permits, DKFZ305). The genetically modified C57Bl6 mouse lines used were *Trp53*^*fl/fl*^*(*abbr. p53*)*, full name B6-Tg(CAG-Brainbow1.0)2Eggn *Trp53*tm1Brn / Aug^38,39^, mouse line ID 3298, Tierbase number 2952, mother B6 *Rainbow2*, father B6 *Trp53*^*fl/fl*^ and *Braf*^*LSL-V600E*^, *Pten*^*lox/lox*^, *Ctnnb1*^*ex3lox/lox*^ (abbr. BPB), full name B6-Braftm1Cpri Ptentm1Hwu Ctnnb1tm1Mmt / Aug^40–42^, mouse line ID 2837, Tierbase number 3340, mother B6 *Braf*^*LSL-V600E*^*Pten*^*lox/lox*^, father B6 *Braf*^*LSL-V600E*^*Pten*^*lox/lox*^*Bcat*^*ex3-lox/lox*^. The mouse lines were originally obtained from the Jackson Laboratory and maintained and bred in the DKFZ animal facility since 2015 with continuous genetic validation (verification primers in Supp. Table 2). The *Trp53*^*fl/fl*^ mouse strain was used since the inactivation of *TP53* is a key mutation in UBC^43^ and has been shown to lead to bladder cancer genesis in mice in conjunction with other mutations but not alone^36^. The BPB mouse strain was used as *CTNNB1*, *PTEN* and *RAS/RAF* pathways all play important roles in UBC genesis in humans^44^. All animal studies were carried out in accordance with the ARRIVE guidelines. No randomisation was used to allocate animals to treatment groups. To minimize confounders, all surgeries were carried out by the same experimenters in the same operation theatre using the same tools and mice were kept in individually ventilated cages in the same animal facility. Animals had access to fresh drinking water and the same nutrition ad libitum. The cages were identical in size, transparent and equipped with regularly changed sawdust and bales of straw or wood for enrichment. Mice were kept in cages by litter in a maximum group size of 6 animals per cage. If animals showed aggressive behavior towards each other, they were set in separate cages. Euthanasia of experimental animals was performed by cervical dislocation.

### Plasmid generation

*Pgk1*-Cre (Addgene plasmid #11543, Klaus Rajewsky, MDC, Berlin), pT-EF1a-KrasG12V, pT-EF1a-Cmyc (Addgene plasmid #92046, Xin Chen, University of California, San Francisco) were provided by Dr. Gürlevik (Hannover), *Pgk1*-SB13 was provided by Dr. Offringa (Heidelberg) and have been previously described^45–51^. *KRT14*-Cre was ordered from and constructed and packaged by vectorbuilder.com (VectorBuilder Inc., USA) using the human *KRT14* promoter. The vectorbuilder ID is VB180515-1200reb, which can be used to retrieve detailed information on vectorbuilder.com. *Cdh1*-Cre was designed using the promoter sequence obtained from the NCBI genome data viewer for the Gene ID 12550 https://www.ncbi.nlm.nih.gov/gene/12550 and sequence identifier NC_000074.6. The 1370 bp upstream of the Mus musculus cadherin 1 gene on chromosome 8 were copied and then also ordered from vectorbuilder.com, the vectorbuilder ID is VB180517-1275dju. *KRT14-*SB13 and *Cdh1*-SB13 were cloned from *Pgk1*-SB13 and *KRT14-*Cre or *Cdh1*-Cre respectively. Plasmid maps and sequence files for the resulting plasmids are attached as supplementary materials. This was done by amplifying the promoter sequences from the respective plasmids via PCR, using NEB Q5^®^High Fidelity DNA Polymerase (New Englang Biolabs, USA), annealing temperatures and primers for PCR (Thermocycler Biometra T3000, Analytik Jena, Germany) were calculated appropriately using the NEBuilder Assembly Tool. The PCR protocol was as follows: Initial denaturation at 98 °C for 30s, followed by 35 cycles of 98 °C for 10s, followed by the specific Tm for primers (KRT14: 65,2 °C, Cdh1: 71 °C) for 30s, followed by 72 °C for 1:30 min and a final extension at 72 °C for 2 min. The following primer sequences were used:

*Cdh1*-SB13 Fwd primer aattcgagctcggtacCCTTTCGAAAGGACCGGGTTCAAATCCCAG;

*Cdh1*-SB13 Rev primer ctcgactctagacttaatCGGGTGCGGTCGGGCAGG;

*KRT14*-SB13 Fwd primer aattcgagctcggtacAAGCTTATATTCCATGCTAGGGTTCTG;

*KRT14*-SB13 Rev primer ctcgactctagacttaatGGTGCAGAGGAGGGAGGTG.

The template plasmids containing the SB13 gene were generated using FastDigest™ (Thermo Fisher Scientific, USA) restriction enzymes PacI (order number FD2204) and KpnI (order number FD0524) following the manufacturer’s protocol. Following agarose gel (agarose from Carl Roth, Germany; ethidium bromide from AppliChem GmbH, Germany; TBE buffer selfmade; electrophoresis ladders from Thermo Fisher Scientific, USA) electrophoresis, plasmid fragments and PCR products were extracted from the gel using the QIAGEN QIAquick Gel Extraction kit (Qiagen, Netherlands), following the supplier’s instructions. Assembly of the new plasmids was done using the NEB Gibson Assembly^®^ Cloning Kit (New England Biolabs, USA), following the manufacturer’s protocol^52,53^.

Bacteria (DH5alpha competent E. coli, Thermo Fisher Scientific, USA) were then transformed using a heat shock transformation protocol (heating block Eppendorf, Germany), seeded onto LB-agar culture plates (standard medium selfmade) containing ampicillin (Sigma-Aldrich, USA) for selection and incubated (Heraeus Function Line B Incubator for Bacteria, Thermo Fisher Scientific, USA) under sterile conditions overnight. Single colonies were picked and placed in standard LB culture medium containing ampicillin. For Giga-preparations of plasmids, the EndoFree^®^ Giga Kit (Qiagen, Netherlands) was used, using between 2,5 L to 8 L of bacterial culture volume (Incubator hood TH30, Edmund Bühler, Germany) and following supplier instructions. All plasmids were verified using adequate primers and the Eurofins genomics services (Eurofins Genomics Europe Shared Services GmbH, Germany) using the following verification primers:

*Cdh1*-SB13 forward primer ATCCCTAAGCAAACAAACTCATCC.

*Cdh1*-SB13 #1 reverse primer TGTACAGATGAACGTGGTACGGC.

*Cdh1*-SB13 #2 reverse primer TTCTTCCTTGCTGAGCGGCCTTTC.

*K14*-SB13 forward primer ACACTCCAAACAATGAGTTTCCAG.

*K14-*SB13 reverse primer TCGCACCAAAGTACGTTCATCTC.

*pUC*-F forward primer GCCAGTGAATTCGAGCTCGG.

*pUC*-R reverse primer TCGCACCAAAGTACGTTCATCTC.

*M13*-RP reverse primer CAGGAAACAGCTATGACC.

*M13*-FP forward primer TGTAAAACGACGGCCAGT.

*pBR1* forward primer CGAAAAGTGCCACCTGAC.

*EF1a*-F forward primer TTTATGCGATGGAGTTTCCC.

### Experimental cohorts

The plasmid solutions applied contained Cre recombinase and SB13 transposase under a general *Pgk1* promoter in the first cohort. In the second cohort, an epithelium-specific *KRT14* or *Cdh1* promoter was used for Cre and SB13 alongside the previous *Pgk1* promoter. Added transposon-based oncogenes in the first cohort were all possible combinations of *KrasG12V* and *cMyc*, i.e., one of the two, both, or neither. In the second cohort, only the combination of both *KrasG12V* plus *cMyc* was used, as this had resulted in the most frequent tumor growth in the first cohort. Some groups had more mice than others due to repeat experiments (e.g., false-positive MRI scans, i.e., suspected tumor in MRI with no evidence of tumor macroscopically). These mice were included in the analyses and statistics. Control groups without treatment were not used, as the aim was to generate tumor growth in the murine bladder, which does not regularly occur in healthy mice. The *Trp53*^*fl/fl*^ group in the first cohort receiving electroporation with a plasmid solution containing only Cre recombinase and SB13 transposase without oncogenes served as a control group for the electroporation process, as according to literature no tumor growth was to be expected by only knocking out *Trp53*^fl/fl^. The experimental unit was a single mouse, n denoting the number of individual mice. We selected a small sample size per group as the aim was to generate a proof of concept that inducing tumor growth is possible while at the same time having sufficient experimental subjects to be able to conclude differences in tumor take rates between different experimental conditions. Pathologists were blinded to the treatment protocol of the samples they received, the experimenters working with animals and plasmids were not blinded and always aware of the group allocation of experimental animals.

### In-vivo electroporation

Postsurgical pain medication was 4 mg/ml metamizole (WDT, Germany) added to the drinking water in conjunction with 16 g/l of glucose (B. Braun, Germany) to enhance taste, thereby ensuring sufficient uptake of pain medication, for 3 days after surgery.

The bladder was then placed between two 5 mm wide platinum covered electroporation electrodes (CUY650P5, NepaGene Co. Ltd., Japan) as shown in Fig. 1B and D. Eight unipolar pulses of 35 V with 50ms pulse length and 950ms of pause with a decay rate of 0% were administered, using the Nepa Gene Super Electroporator NEPA21 Type II (NepaGene Co. Ltd., Japan). The bladder was then repositioned, the abdominal wall was sutured in two steps and the wound area was covered with iodine solution to prevent infection. Animals were injected subcutaneously with sodium chloride as appropriate to prevent dehydration as well as 200 mg/kg BW metamizole (WDT, Germany) for pain medication, placed in a clean cage on a warming mat for waking up and observed for any signs of clinical deterioration following surgery.

For in vivo electroporation, mice (10–14 weeks old) were anesthetized using Ketamin (100 mg/kg BW) (Ketavet, Pfizer, USA) and Xylazin (10 mg/kg BW) (Rompun, Bayer, Germany) administered via intraperitoneal injection. Efficacy of anesthesia was tested via the foot pinch reflex and eyes were covered in Bepanthen eye cream (Roche, Switzerland) to prevent drying out. The lower abdomen was then shaved and sterilized (veterinary alcoholic iodine solution, WDT, Germany), the operation site and all tools were sterilized and the operation was carried out under sterile conditions. Supplementary Fig. 1 shows the full surgical procedure. A short incision starting directly above the pelvis was made and the skin was mobilized from the peritoneum using the blunt ends of scissors. Then a small opening in the peritoneum was created, the bladder was identified and mobilized. In the first experimental cohort, both male and female mice were employed and treated the same: 50–100 µl of plasmid solution (concentration 8–10 µg/µl) was injected using a 27G injection needle. In the second cohort, catheterization was used for females, i.e., a urinary catheter (outer diameter 0.61 mm, inner diameter 0.28 mm) was inserted through the urethra into the bladder and used to instill the plasmid solution. Male mice were treated using transmural injection.

### Post-interventional monitoring

#### Animals were closely monitored following surgery with daily clinical examination

For the MRI imaging, mice were anesthetized using 2,5% v/v Sevoflurane (B. Braun, Germany) using the vaporizer Dräger Vapor 2000 (Drägerwerk & Co. KG, Germany). MRI imaging (MRI imager ICON 1 Tesla and MRI imager 3 Tesla BiosSpec 3T, Bruker BioSpin GmbH, Germany) of the lower abdomen was initially done every 2 weeks and, upon adjustment of experimental protocol and animal permit, up to twice a week in cases of signs of tumor growth based on previous imaging results or clinical examination.

### Endpoints

Mice were euthanized immediately upon reaching any of the pre-defined humane endpoints or clear evidence of tumor growth. Humane endpoints for all experiments were weight loss above 20%, apathy or abnormal movement or body position indicating pain, insufficient uptake of food and water, gross inflammation or necrosis in the wound area or at injection sites, tumor ulceration, certain tumor detection or urinary obstruction in MRI examination, macrohematuria and signs of bad clinical regeneration after surgery. A necropsy was carried out and tumor tissue was extracted and processed for further analysis as described below. Mice without tumor growth at reaching a humane endpoint were still included in the analysis and included as censored in the tumor take rate curves. Other than the age of 10–14 weeks, no exclusion criteria were applied before the operation and electroporation.

### Histology, staining and pathological analysis

Histology samples were placed in Zinc-fixative solution (1 M Tris-HCl diluted 1:10 with deionized water and set to pH 7.4 using NaOH, then addition of 5 g/l zinc-acetate-dihydrate, 5 g/l zinc-chloride and 0.5 g/l calcium-acetate-hydrate under constant stirring) at the time of necropsy for a maximum of 48 h and later embedded in paraffin following standard procedures using a tissue processing carousel and paraffin embedding machine (embedding machine for paraffin tissues Microm EC 350-1 and EC 350-2 and tissue processor STP 120 Spin Tissue Processor, both Thermo Fisher Scientific, USA). Tissue sections were deparaffinized and rehydrated, following standard protocol using HistoClear II (Linaris, Germany) and a descending alcohol solution. For Hematoxylin-Eosin staining, standard protocol was followed, using Mayer’s hemalaun solution (Sigma-Aldrich, USA) for 4 min, rinsing with tap water for 10 min and 2 min in eosin solution (Agilent Dako, USA), before re-dehydration and mounting with HistoMount solution (Thermo Fisher Scientific, USA) and a cover glass. For IHC staining, antigen retrieval was carried out after rehydration, using citric acid buffer (Agilent Dako, USA) at pH 6.0 for 40 min in a water bath at 97 °C, followed by washing. Peroxidase blocking using 3% H2O2 solution (Th. Geyer, Germany) for 15 min, protein blocking using 1% BSA (Gerbu, Germany) and 5% goat serum (Agilent Dako, USA) for 1 h at RT and avidin and biotin blocking (Agilent Dako, USA) for 15 min each were carried out separated by washing steps to optimize results. Primary antibody diluted in blocking solution was applied overnight at 4 °C. After several washing steps, biotinylated secondary antibody (order numbers BA-1000, BA-9401, BA-9400, all from Vectorlabs, USA) was applied at RT for 1 h. Avidin-Biotin solution (Vectastain Elite ABC kit PK-6100, Vectorlabs, USA) was prepared following supplier protocol and applied after another washing step. Slides were developed individually by adding di-amino-benzidine (DAB) (DAB peroxidase kit SK-4100, Vectorlabs, USA) and a set time before washing off excess DAB. After counterstaining with Mayer’s hemalaun for 4 min and tap water rinsing for 10 min, slides were dehydrated and mounted as described above.

Primary antibodies used were: anti-pancytokeratin by Bioss USA, order number bs-1712R diluted 1:200; anti-Upk3 by Abcam order number ab231576 diluted 1:100; anti-vimentin by Cell signaling order number 57415 diluted 1:100; anti-CK5 Abcam order number ab53121 diluted 1:200. The secondary antibody used was a biotinylated anti-rabbit IgG by Vectorlabs order number BA-1000 diluted 1:500.

Both H.E. and IHC staining slides were scanned using the AxioScanZ.1 slide scanner (Carl Zeiss AG, Germany) running ZEN 2 (blue edition) software, not processed any further with imaging software and sent digitally or as glass slides for independent analysis by two pathologists (C.M., M. E.) blinded to the experimental conditions of each slide. Images for publication were extracted directly from the slide scanner software ZEN 2 (blue edition) and not modified.

### RNA extraction and RT-qPCR

Tissue samples for RNA analysis were snap-frozen using liquid nitrogen at the time of autopsy and stored at -80 °C until further processing. Tissue parts were placed in a reaction tube with 500 µl of Trizol reagent (Invitrogen, Thermo Fisher Scientific, USA) and a metal pellet and put in a shaking mill at a frequency of 30/s for 1 min until complete lysis. This was followed by adding 100 µl of chloroform (VWR chemicals International, Germany), 30s of vortexing and 5 min of incubation at room temperature before centrifugation at 15.000 g for 15 min at 4 °C. The top layer containing the RNA was pipetted into a new reaction tube with the same amount of 100% ethanol for precipitation and vortexed briefly. RNA purification was done using the GenElute^™^ Mammalian Total RNA Purification Kit (Kit RTN 350, Sigma Aldrich, USA), following the manufacturer’s instructions. RNA concentration and purity was subsequently measured using a Nanodrop N60 photometer (Implen, Germany) with measurements for 230 nm, 260 nm and 280 nm absorption and ratios. Samples with A260/280 ratios below 2.0 or A260/230 ratios below 2.0 were discarded. RNA was either stored at -80 °C or instantly transcribed into cDNA. cDNA generation from RNA was done using the QuantiTect^®^ Reverse Transcription Kit (Kit 205313, Qiagen, Netherlands) following the supplier manual and using 1000ng of pure RNA. cDNA was stored at -20 °C until use for qPCR analysis.

Real time quantitative PCR (RT-qPCR) was carried out with TaqMan™ RT-qPCR (Applied Biosystems, Thermo Fisher Scientific, USA) assays following supplier instructions. 4 µl of a 1:20 diluted cDNA solution resulting from 1 µg of RNA were used per 10 µl qPCR reaction volume, consisting of 5 µl TaqMan™ Fast Advanced Mastermix, 0.5 µl of nuclease-free water and 0.5 µl of the gene-specific TaqMan™ probe. PCR and analysis was done using the LightCycler^®^ 480 (Roche, Switzerland), using following protocol: 20s at 95 °C for denaturation followed 45 cycles of 2s at 95 °C for denaturation and 20s at 60 °C for synthesis and detection after every cycle.

Gene expression levels were analyzed using the 2^ΔΔCT^ method^54,55^. All gene expression analyses shown are given relative to the expression levels in healthy mouse bladder tissue taken from animals with no genetic modification for normalization. We identified the 2 most stable housekeeping genes to be *Hprt1* and *Tbp* using Normfinder as described by Andersen et al.^56^ and used a geometric average of the CT of *Hprt1* and *Tbp* values as the reference gene value as shown by Vandesompele et al.^57^.

### Analysis and statistics

Statistical analysis was done using the software GraphPad Prism 5 (GraphPad Software Inc., USA). Levels for statistical significance were set at *p* < 0.05 for *, *p* < 0.01 for ** and *p* < 0.001 for ***. Appropriate statistical tests were chosen depending on the data analyzed. Comparison of two groups was done using the Mann-Whitney test as a non-parametric t-test due to the small group sizes. Where more than 2 groups were compared, repeated Mann-Whitney tests with Bonferroni correction (correction for *p* < 0.05 / number of groups n) were applied. Kaplan-Meier curves were generated for tumor take (i.e. also point of sacrifice), including censored points at the point of sacrifice in the case of necropsy without evidence of tumor, and analyzed using log-rank comparison / Mantel-Cox test.

## Electronic supplementary material

Below is the link to the electronic supplementary material.

Supplementary Material 1

## Data Availability

All data necessary to replicate and use the presented model is included in the article. Raw data files containing individual qPCR data, listing pathological diagnoses for each mouse or listing experimental details and outcomes for all individual mice are available via Email upon reasonable request. Likewise, all pathological images in original resolution can be made available on a hard drive disk upon reasonable request upon sending of a hard drive of sufficient size (1 TB) and coverage of return shipping expenses. Requests should be addressed to Stefan Soleder at stefan.soleder@gmail.com.
