# Supplementary material for: Development of a novel immunocompetent murine tumor model for urothelial carcinoma using in vivo electroporation
Source: Sci Rep. 2024 Oct 27;14:25619. doi: 10.1038/s41598-024-77178-z (PMC11514157; doi:10.1038/s41598-024-77178-z)
Supplement: Supplementary file 1 — Supplementary Material 1 [file 41598_2024_77178_MOESM1_ESM.pdf]

SUPPLEMENTARY MATERIAL

Development of a novel immunocompetent murine tumor model for urothelial carcinoma using in vivo electroporation

Stefan Soleder, Nicolas Gengenbacher, Carolin Mogler, Markus Eckstein, Anja Runge, Maximilian C. Kriegmair, Hellmut G. Augustin

Supplementary Material

Supplementary figure 1

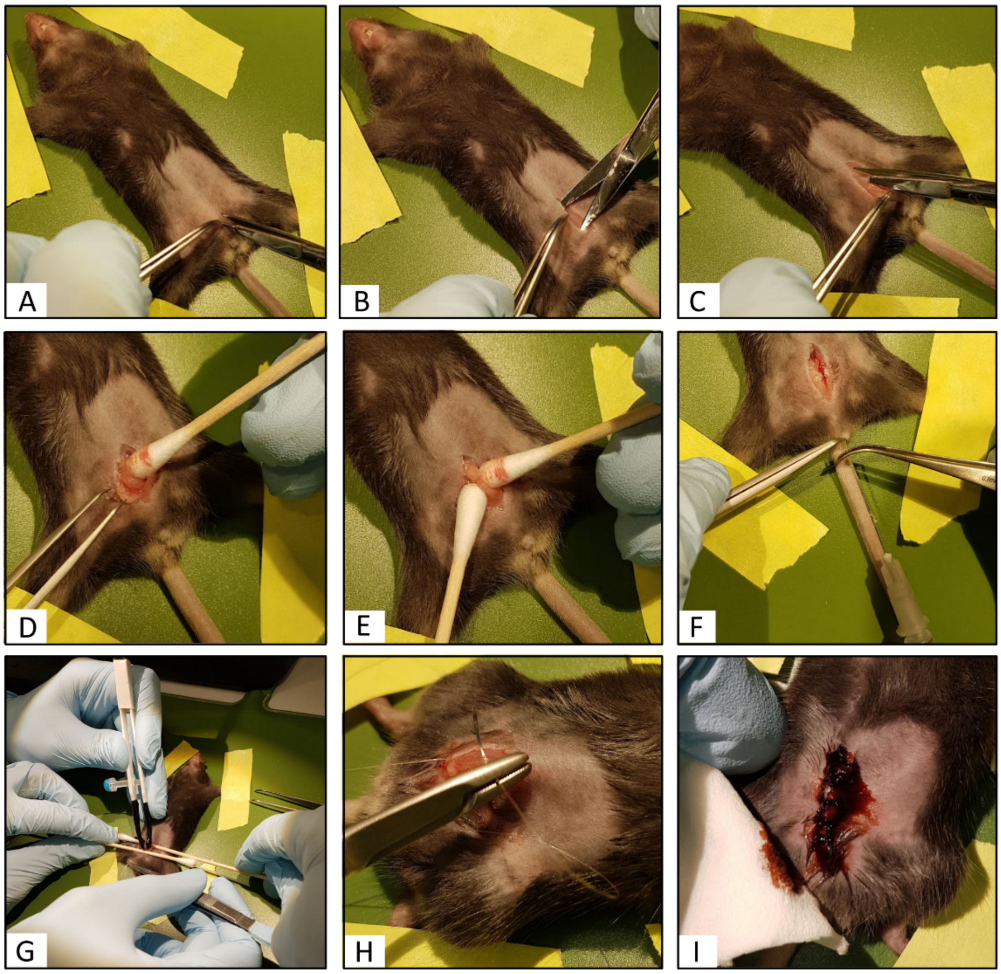

Suppl. Fig. 1: Complete surgical procedure, catheterization and electroporation. A-C show skin incision, mobilization of peritoneum from the skin and peritoneal incision, D-E show mobilization of the bladder with tweezers and a cotton swab, squeezing to empty urine and repositioning, F shows catheterization with attached syringe containing plasmids (male bladders injected with a needle), G shows electroporation carried out by two experimenters, one stabilizing the filled bladder and one operating electrodes and electroporator, H-I show suturing of peritoneum and skin and application of iodine solution

Supplementary figure 2

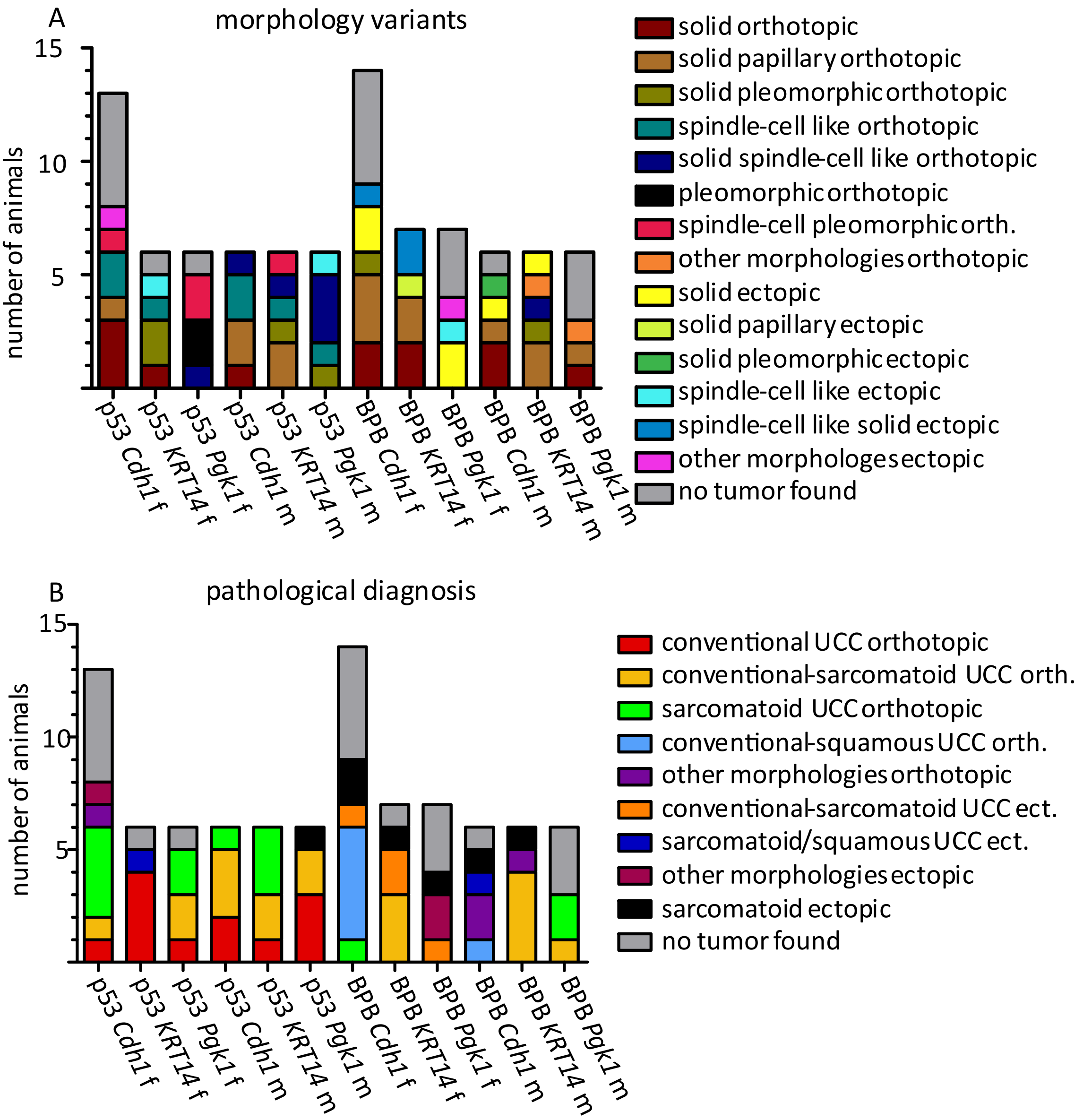

Suppl. Fig. 2: Histopathological diagnosis and morphological patterns show different variants of mostly urothelial cell carcinomas. A shows a detailed morphological analysis of the primary and secondary growth patterns of the resulting tumors; B shows the resulting diagnosis based on HE- and IHC-stainings.

Supplementary Figure 3

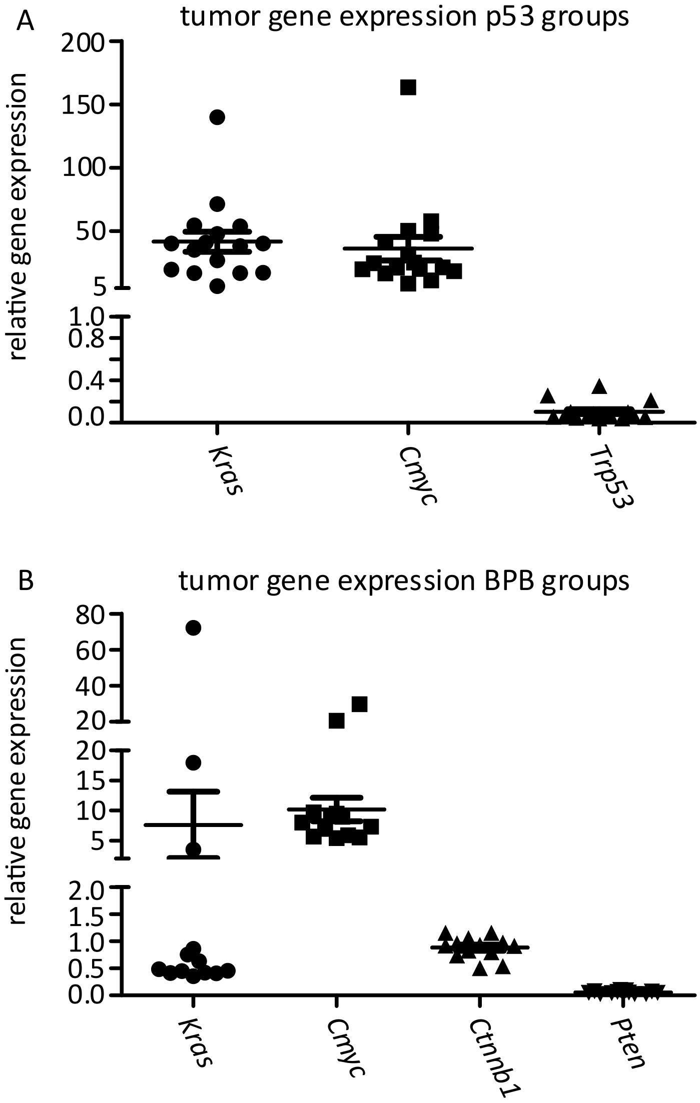

Suppl. Fig. 3: Relative gene expression of inserted or knocked-out genes. A shows overexpression of *Kras* and *Cmyc* and knockout of *Trp53* in the p53 background tumors; B shows normal levels of *Ctnnb1* (mutation caused by Cre is removal of exon 3, not knock-out) and knockout of *Pten*, overexpression of *Cmyc*, varying levels of gene expression of *Kras* in the BPB background tumors .

Supplementary Table 1

Supplementary Table 1: High rates of orthotopic urothelial cell carcinomas in most groups, rapid tumor development and high rates of metastasis across all tumors.

experimental group	n	median time to detection, **any kind of tumor**	median time to detection, **orthotopic UCC tumor**	% of mice with **any kind of tumor**	median time to sacrifice, **mice without tumor**	% of mice with **ortho-topic UCC tumor (success)**	% of orthotopic UCC **with metastasis**	% of orthotopic UCC **with muscle invasiveness**
**first cohort, no differentiaton by sex (all direct injection)**								
p53 *Pgk1-Cre*	6	no tumors	no tumors	0 %	300 d	0%	-	-
p53 *Pgk1-Cre + Kras*	5	44 d	40 d	100 %	-	80 %	50 %	50 % (-75 %)
p53 *Pgk1-Cre + Cmyc*	5	83 d	83 d	80 %	44 d	40 %	100 %	50 % (-100 %)
p53 *Pgk1-Cre + Kras + Cmyc*	6*	29 d	29 d	83,3 %	After OP	83,3 %	20 %	40 % (-80 %)
BPB *Pgk1-Cre*	8	51 d	51 d	62,5 %	280,5 d	62,5 %	60 %	60 % (-100 %)
BPB *Pgk1-Cre + Kras*	6	69 d	69 d	100 %	-	66,7 %	25 %	25 % (-100 %)
BPB *Pgk1-Cre + Cmyc*	6	43 d	44 d	100 %	-	83,3 %	80 %	20 % (-100 %)
BPB *Pgk1-Cre + Kras + Cmyc*	6	55 d	59 d	83,3 %	59 d	16,6 %	0 %	0 % (-100 %)
** 1 mouse euthanized right after OP*								
**second cohort, sexes with differing treatment protocol (catheterization for females)**								
p53 female (catheterization)								
p53 *Cdh1-Cre/SB13 + Kras + Cmyc*	13	26 d	26 d	61,5 %	50 d	53,8 %	71,4 %	71,4 % (-85,7 %)
p53 *KRT14-Cre/SB13 + Kras + Cmyc*	6	26 d	26 d	83,3 %	29 d	66,7 %	50 %	75 %
p53 *Pgk1-Cre/SB13 + Kras + Cmyc*	6	25 d	25 d	83,3 %	35 d	83,3 %	20 %	60 % (- 100 %)
p53 male (direct injection)								
p53 *Cdh1-Cre/SB13 + Kras + Cmyc*	6	22,5 d	22,5 d	100 %	-	100 %	66,7 %	80 % (-100 %)
p53 *KRT14-Cre/SB13 + Kras + Cmyc*	6	23 d	23 d	100 %	-	100 %	83,3 %	100 %
p53 *Pgk1-Cre/SB13 + Kras + Cmyc*	6	20 d	20 d	100 %	-	83,3 %	40 %	80 % (-100 %)
BPB female (catheterization)								
BPB *Cdh1-Cre/SB13 + Kras + Cmyc*	14	35 d	30 d	64,3 %	57 d	42,9 %	100 %	80 %
BPB *KRT14-Cre/SB13 + Kras + Cmyc*	7	37,5 d	42 d	85,7 %	48 d	42,9 %	66,7 %	66,7 %
BPB *Pgk1-Cre/SB13 + Kras + Cmyc*	7	23,5 d	-	57,1 %	29 d	0 %	-	-
BPB male (direct injection)								
BPB *Cdh1-Cre/SB13 + Kras + Cmyc*	6	30 d	48 d	83,3 %	63 d	16,7 %	100 %	100 %
BPB *KRT14-Cre/SB13 + Kras + Cmyc*	6	30 d	30 d	100 %	-	66,7 %	100 %	100 %
BPB *Pgk1-Cre/SB13 + Kras + Cmyc*	6	30 d	30 d	50 %	30 d	50 %	33 %	66,7 % (-100 %)
2 mice euthanized right after OP	2							

Supplementary Table 2

Supplementary Table 2: Mouse strain verification RT-qPCR primers used.

Genotype	Primer	Sequence (5'-3')
Rainbow2 x p53flox	Rainbow2 for<br>Rainbow2 rev<br>p53 floxed for<br>p53 floxed rev	GGCACGCTGATCTACAAGGT<br>GGGAGGTGTGGGAGGTTTT<br>CACAAAAAACAGGTTAAACCCA<br>AGCACATAGGAGGCAGAGAC
BrafLSL-V600E x PTEN-lox x Bcat-ex3-lox	Braf CA for<br>Braf CA rev<br>Pten for<br>Pten rev<br>Pten CPRB<br>Bcat-Intr2 Fwd-F<br>Bcat-Intr2 R175-R	TGAGTATTTTTGTGGCAACTGC<br>CTCTGCTGGGAAAGCGGC<br>TGTCTGGCAATGCTGTAGTAATA<br>AAGATAATCCCAGTGTAAGAAAA<br>GCATACATTATACGAAGTTATGGC<br>GATGCCTGTCTGAGGATCTGC<br>CAGGTGAGGGTCAGTATGAGC
